# Immunohistochemical characterization of tuberculous lesions in sheep naturally infected with *Mycobacterium bovis*

**DOI:** 10.1186/s12917-018-1476-2

**Published:** 2018-05-04

**Authors:** Raquel Vallejo, Juan Francisco García Marín, Ramón Antonio Juste, Marta Muñoz-Mendoza, Francisco Javier Salguero, Ana Balseiro

**Affiliations:** 1 Universidad de León, Campus de Vegazana, León, Spain; 2 SERIDA, Servicio Regional de Investigación y Desarrollo Agroalimentario, Centro de Biotecnología Animal, 33394 Gijón, Asturias Spain; 3 Xunta de Galicia, Santiago, A Coruña, Galicia Spain; 4 School of Veterinary Medicine, University of Surrey, Guildford, UK

**Keywords:** Tuberculosis, Sheep, Immunohistochemistry, *Mycobacterium bovis*, Granuloma

## Abstract

**Background:**

Sheep have been traditionally considered as less susceptible to *Mycobacterium bovis* (Mbovis) infection than other domestic ruminants such as cattle and goats. However, there is increasing evidence for the role of this species as a domestic Mbovis reservoir, mostly when sheep share grazing fields with infected cattle and goats. Nevertheless, there is a lack of information about the pathogenesis and the immune response of Mbovis infection in sheep. The goals of this study were to characterize the granuloma stages produced by the natural infection of Mbovis in sheep, to compare them with other species and to identify possible differences in the sheep immune response. Samples from bronchial lymph nodes from twelve Mbovis-naturally infected sheep were used. Four immunohistochemical protocols for the specific detection of T-lymphocytes, B-lymphocytes, plasma cells and macrophages were performed to study the local immune reaction within the granulomas.

**Results:**

Differences were observed in the predominant cell type present in each type of granuloma, as well as differences and similarities with the development of tuberculous granulomas in other species. Very low numbers of T-lymphocytes were observed in all granuloma types indicating that specific cellular immune response mediated by T-cells might not be of much importance in sheep in the early stages of infection, when macrophages are the predominant cell type within lesions. Plasma cells and mainly B lymphocytes increased considerably as the granuloma developed being attracted to the lesions in a shift towards a Th2 response against the increasing amounts of mycobacteria. Therefore, we have proposed that the granulomas could be defined as initial, developed and terminal.

**Conclusions:**

Results showed that the study of the lymphoid tissue granulomata reinforces the view that the three different types of granuloma represent stages of lesion progression and suggest an explanation to the higher resistance of sheep based on a higher effective innate immune response to control tuberculosis infection.

## Background

Recent studies have shown that sheep, traditionally considered a rare host for the *Mycobacterium tuberculosis* complex (MTBC), can be part of the multi-species system which can maintain tuberculosis (TB) in a region, at least in mixed farms where sheep share grazing fields with *Mycobacterium bovis* (Mbovis)-infected cattle and goats [1]. Microscopic lesions in sheep infected with Mbovis are characterized by localized and well delimited granulomas mainly in lungs and bronchial lymph nodes [1], although limited information has been published about the pathogenesis and immune response in sheep against TB [2–4].

Granulomas are observed in active, latent and reactivation stages of TB. The TB lesion is highly dynamic and shaped both by the pathogen and the host defense elements [5]. In humans generally the TB granuloma successfully contains (but does not eliminate) the infectious focus in more than 90% of cases, whereas 10% of individuals progresses towards clinical TB as a consequence of an unbalanced inflammatory reaction. The granuloma is capable of limiting growth of mycobacteria but also is a good environment from which the bacteria may disseminate [5]. Immunohistochemical techniques are valuable tools to advance the knowledge of the immunology of infection for a better understanding of the mechanisms of host-pathogen interactions and disease progression, as has been reported in Mbovis-infected cattle, badger, fallow deer and wild boar [6–10].

To gain further insight into the knowledge of the immunopathology of tuberculous lesions in sheep, the goals of this study were to characterize the granuloma stages produced by the natural infection of Mbovis in sheep, to compare them with other species and to identify possible differences in the sheep immune response.

## Methods

### Samples

A total of 12 sheep naturally infected with Mbovis (spolygotype SB0886) from the Galicia region (Northwestern Spain) were selected from a previous study [1]. In the former study at post mortem examination, small granulomatous nodules of less than 5 cm in diameter were histologically classified in stages I, II and III on the basis of cell inflammatory influx, inflammatory cell type, mineralization and calcification and degree of encapsulation. Briefly, type I were unencapsulated granulomas consisted mainly of epithelioid cells, macrophages, lympochytes and few Langhan’s multinucleated giant cells in which sometimes a minimum central focus of necrosis was observed. Type II were granulomas (often diffuse) composed of numerous inflammatory cells, mainly macrophages and Langhan’s giant cells surrounded by a full thin fibrotic capsule with central necrotic areas which were caseous or partly calcified. Finally, type III were well encapsulated granulomas with areas of central caseous necrosis with mineralization occupying the majority of the lesion, surrounded by scarce inflammatory infiltrate consisted mainly of lymphocytes. Ziehl-Neelsen stain was used to identify and count acid fast bacilli (AFBs) within lesions in four random 400× magnification fields.

Twelve sheep showing granulomas only in bronchial lymph nodes were selected, four sheep with granuloma type I, four with granuloma type II and four with granuloma type III. We choose the bronchial lymph nodes because they were the most frequently affected tissues [1]. They were used for the immunohistochemical characterization of cellular types. For each tissue section and to equilibrate counts, the sum of four randomly selected different areas of 400× magnification were analyzed using a light microscope in order to count the total number of cells immunostained for each cell type. Based on the number of total cellular type counted, a semi-quantitative classification was elaborated as follows: (−) abscence of immunolabelled cells; (+) 1–10 immunolabelled cells; (++) 11–50 immunolabelled cells; (+++) 51–100 immunolabelled cells; (++++) >  100 immunolabelled cells.

### Immunohistochemistry (IHC)

Four-μm sections were used for immunohistochemical detection of four different antigens (Table 1). The ABC Complex reagent-method (Vector Laboratories, California, USA) was used. Briefly, the sections were deparaffinised, rehydrated and rinsed with tap water. Afterwards, slides were treated to quench the endogenous peroxidase by incubation with methanol containing 3% H_2_O_2_ for 10 min at room temperature (RT) and washed with water for 10 min. Then, epitope demasking techniques (Table 1) were used to retrieve the antigens and samples were treated to prevent unspecific binding with a 20 min incubation at RT with 10% normal horse serum for CD3 protocol (DAKO, Glostrup, Denmark) or with 10% normal goat serum for CD20, Iba1 and Lambda protocols, and 3% bovine serum albumin (BSA) in tris buffered saline (TBS, 5 mM Tris/HCl pH 7.6, 136 mM NaCl). The tissue sections were incubated overnight at 4 °C with commercial monoclonal and polyclonal antibodies (Table 1) and then washed three times with TBS. Then, samples were incubated with horse anti-mouse serum or goat anti-rabbit serum (Table 1) (Vector Laboratories, California, USA) diluted 1:200 in TBS for 30 min at RT and washed three times with TBS followed by incubation with the ABC complex kit in TBS for 30 min at RT. Finally, the sections were incubated with the substrate 3,3′-diaminobenzidine tetrahydrochloride (DAB, Sigma, St. Louis, MO, USA) for 5 min and washed with TBS and water. After staining for 45 s with haematoxylin, slides were dehydrated and mounted with DPX (Fluka, Sigma, St. Louis, MO, USA). Stained slides were studied under light microscopy (Olympus BH-2) and photographed using a digital camera Olympus DP-12. Positive (where the target antigen was present in the control section and the specific antibody was used) and negative (additional slide with omission of the primary antibody) controls were used during each immunohistochemical run.

**Table 1:** Immunohistochemical protocols used for cellular type characterization

Primary antibody (Ab)	Specifity	Dilution Ab	Epitope demasking	Secondary Ab
CD3 (Novocastra-CL-L-CD3–565), mouse monoclonal	Pan T cell marker	1:500 in TBS 1%	Microwave in citrate pH 6 20 min	Anti-mouse biotinylated (1:200)
CD20 (ThermoFisher.-PA516701), rabbit polyclonal	Pan B cell marker	1:200 in TBS + BSA 1%	Microwave in citrate pH 6 20 min	Anti-rabbit biotinylated (1:200)
Iba1 (WAKO 019_19741), rabbit polyclonal	Macrophages	1:1000 in TBS + BSA 1%	Los Angeles pH 9 40 min 95 °C	Anti-rabbit biotinylated (1:200)
Lambda (Dako A0193), rabbit polyclonal	Plasma cells	1:1000 in TBS + BSA 1%	Triton 1% 20 min room temperature	Anti-rabbit biotinylated (1:200)

*TBS* Tris-buffered saline, *BSA* Bovine serum albumin

### Statistical analysis

Results were submitted to analysis of variance to determine the statistical significance of differences between granuloma types for each cell type and then the means were compared with the Tukey-Kramer test in the SAS statistical package.

## Results

The number of AFBs identified in each type of granuloma was very low in three types of granulomas (< 10 AFBs in type I granuloma and 10–20 AFBs in types II and III granulomas) and no differences were observed between them.

Significant differences in the numbers of cell types were observed in each TB granuloma type (Table 2).

**Table 2:** Cellular types found in tuberculous granulomas

	Initial granuloma	Developed granuloma	Terminal granuloma
T lymphocytes	+	+	–
B lymphocytes	++	+++	++++
Macrophages	+++	++++	++
Plasma cells	–	++	+++

-: abscence of immunolabelled cells; +: 1–10 immunolabelled cells; ++: 11–50 immunolabelled cells; +++: 51–100 immunolabelled cells; ++++: > 100 immunolabelled cells

### CD3 (T lymphocytes)

**Fig. 1:**
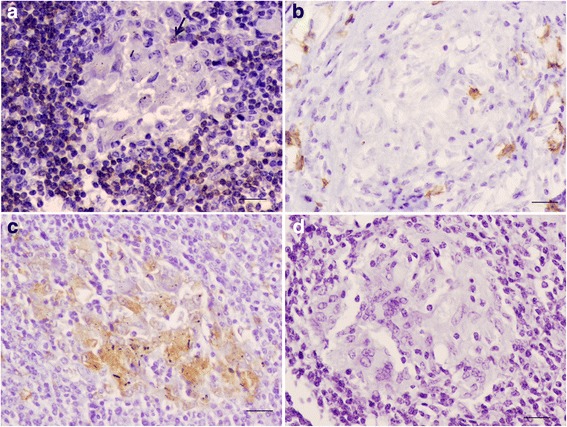
Bronchial lymph node; immunohistochemical characterization of cellular populations in initial granuloma. (**a**) Isolated T lymphocytes (arrow) can be observed in the granuloma, bar = 20 μm. (**b**) A few scattered B lymphocytes are observed in this stage, bar = 20 μm. (**c**) Abundant immunolabelled macrophages are present, bar = 20 μm. (**d**) No plasma cells are observed, bar = 20 μm

**Fig. 2:**
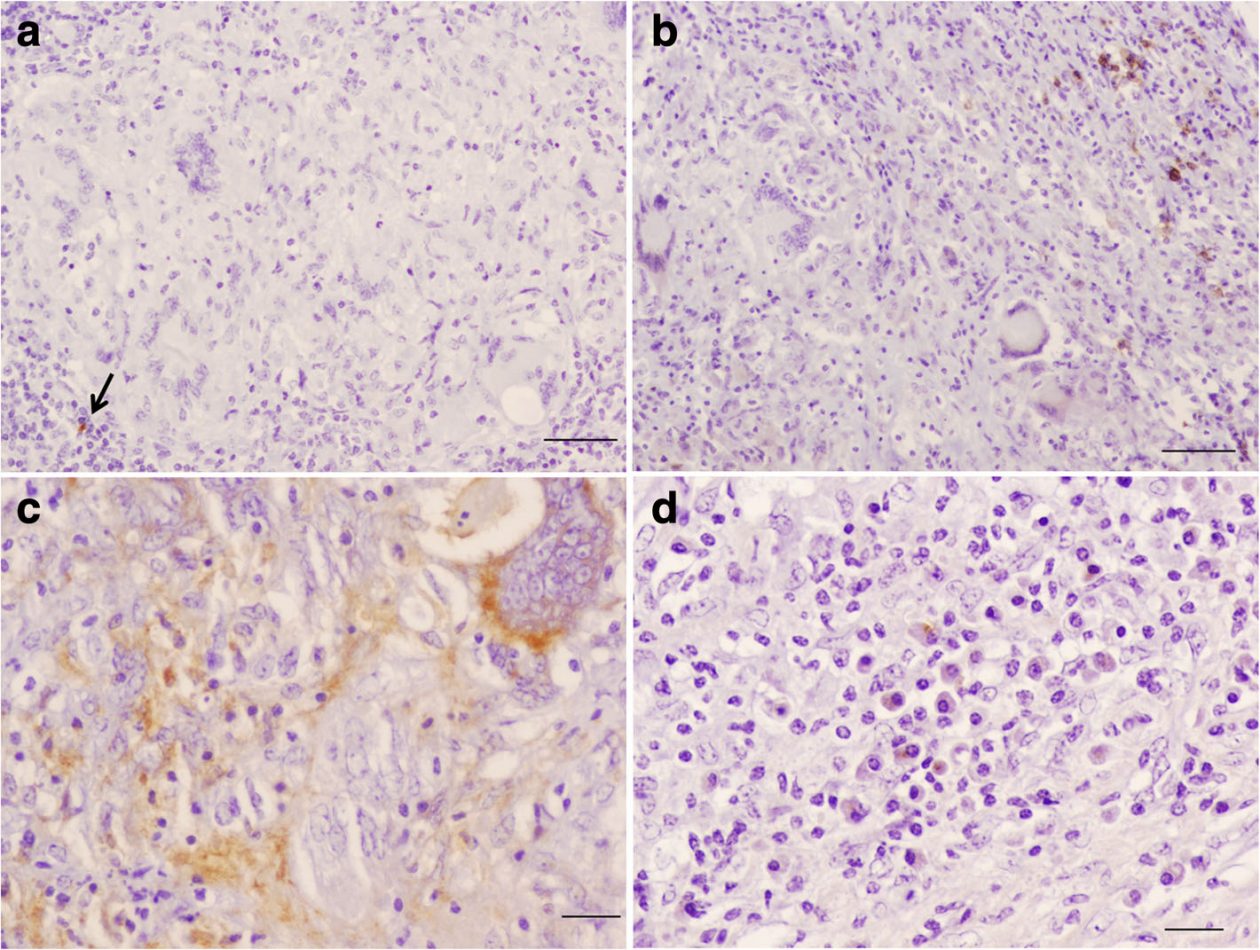
Bronchial lymph node; immunohistochemical characterization of cellular populations in developed granuloma. Scattered T (arrow) and B lymphocytes are observed towards the periphery of the granuloma (**a** and **b**, bar = 50 μm). The presence of immunolabelled macrophages predominated over the whole granuloma (**c**, bar = 20 μm), while plasma cells were abundant and peripherally located (**d**, bar = 20 μm)

**Fig. 3:**
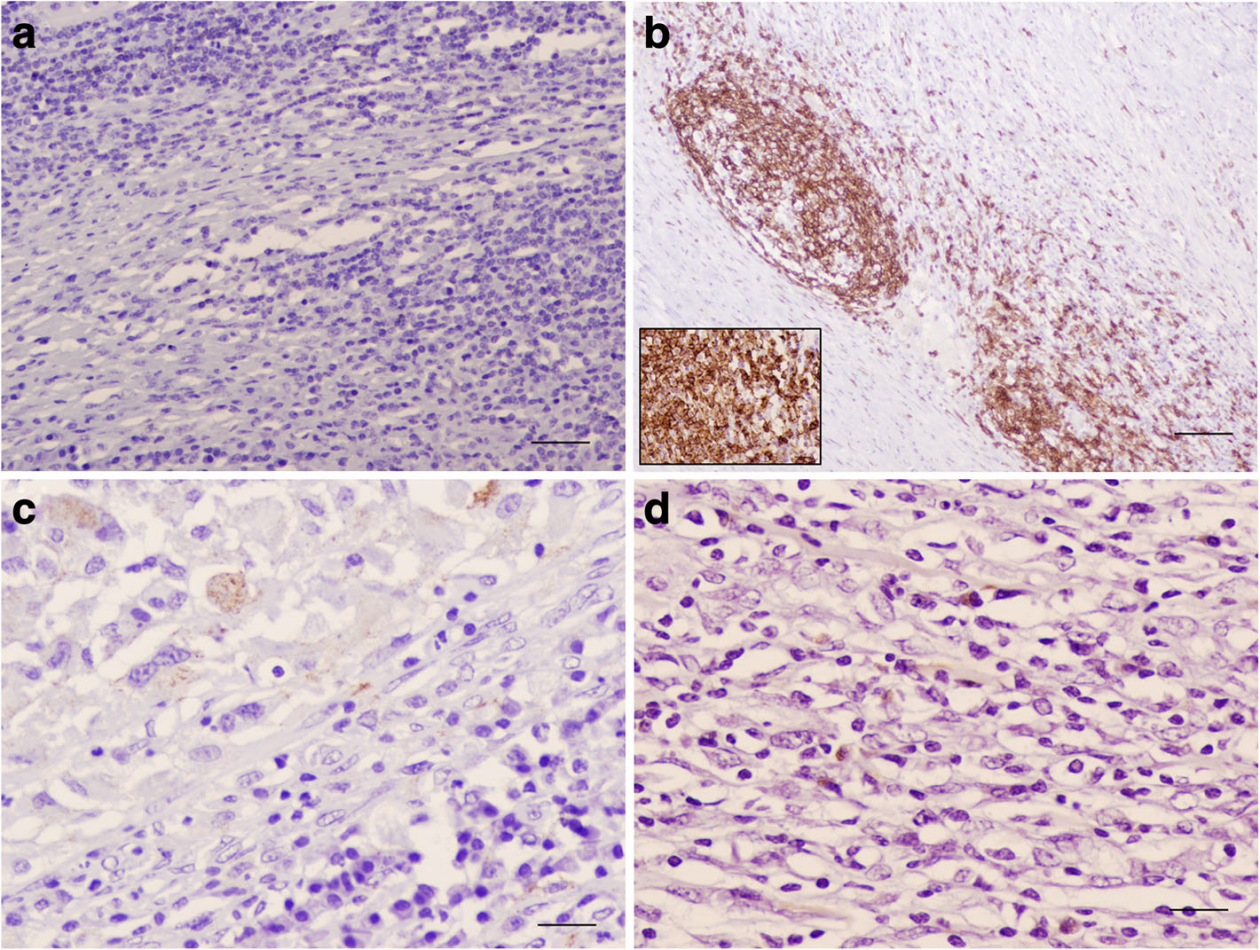
Bronchial lymph node; immunohistochemical characterization of cellular populations in terminal granuloma. No T lymphocytes were observed within the granuloma (**a**, bar = 20 μm). The main cellular type were B lymphocytes forming clusters surrounding the necrotic area of the granuloma (**b**, bar = 200 μm; inset: bar = 20 μm). Positive immunolabelled macrophages and plasma cells were randomly and sparsely distributed (**c** and **d**, bar = 20 μm)

IHC analysis for CD3 showed very few scattered positively stained lymphocytes within type I and type II granulomas (Table 2, Figs. 1 and 2), randomly and peripherally distributed, respectively (Figs. 1 and 2), with no T cells observed within type III granuloma (Fig. 3).

### CD20 (B lymphocytes)

B cells were found in all granulomas, mainly in type III granulomas, forming clusters surrounding the necrotic area (Fig. 3). In types I and II granulomas, these cells were sparsely distributed within the lesion.

### Iba1 (macrophages)

Macrophages were the predominant cells observed within granulomas of the types I and II (Figs. 1 and 2). However, the number of macrophages decreased for the type III granuloma type and appeared peripherally distributed (Fig. 3).

### Lambda chain (plasma cells)

No cells immunostained for lambda chains were observed in type I granuloma (Fig. 1). A few sparsely distributed cells at the periphery of the types II and III granulomas were observed (Figs. 2 and 3).

### Statistical results

**Fig. 4:**
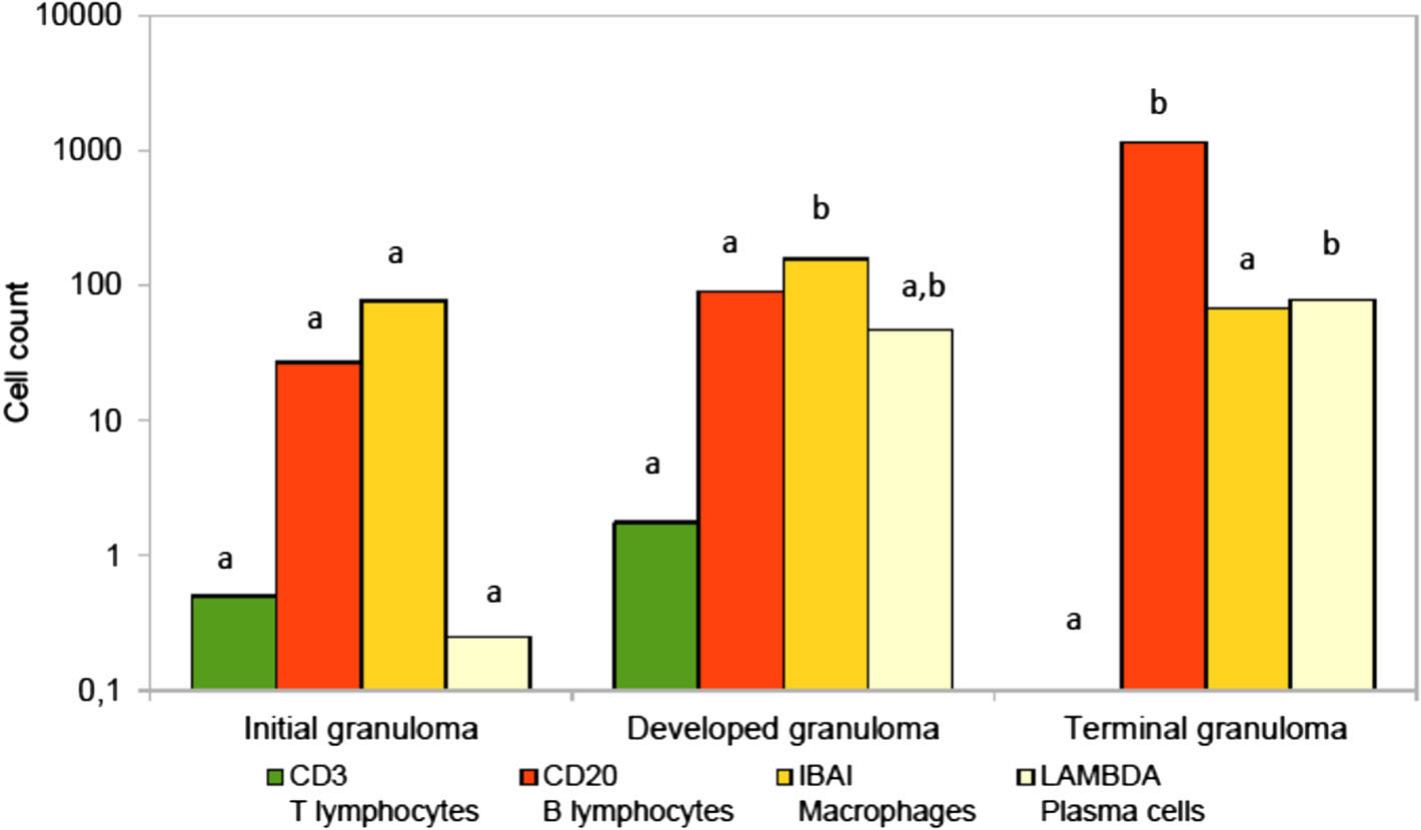
Comparison between mean cell type counts in tuberculous granulomas. Cell counts are represented in a logarithmic scale. Cell type is identified both after the specific marker and the cell type name. Different letters indicate significant differences at *p* < 0.05 between granuloma type. No differences between granulomas for T lymphocyte marker; greater mean for B lymphocyte marker in terminal granulomas; greater mean for macrophages in developed granulomas than in initial granuloma, but no differences for the other comparisons; greater mean for plasma cells in terminal granuloma

Significant differences in the mean number of cells were found for B lymphocytes and plasma cells (*p* < 0.0001), but not for T lymphocytes and macrophages (Fig. 4). The clearest differences were in the number of B lymphocytes that were observed at a different order of magnitude in each granuloma type. Plasma cells only showed differences between types I and III granulomas. In summary, the B lymphocyte was the cell that best discriminated between the three granuloma subtypes.

Therefore according to their cell composition we defined tuberculous granulomas as *initial* (type I), *developed* (type II) and *terminal* (type III). *Initial* granuloma would represent an initial or latent stage, *developed* granuloma a more mature stage and *terminal* granuloma a regressive stage, associated with mineralization and fibrosis indicative of resolution.

## Discussion

This preliminary study describes, for the first time, the distribution of T cells, B cells, macrophages and plasma cells in tuberculous granulomas from sheep naturally infected with Mbovis. Differences were observed in the predominant cell type present in each type of granuloma, as well as differences and similarities with the development of tuberculous granulomas in other species [6–10].

It is important to note the scarce presence of T lymphocytes in all granuloma types (< 10 cells), which would suggest a clear difference with the spectrum of lesions of TB in other species, such as cattle, where T lymphocytes are essential in the initial cellular immune response against Mbovis [6, 7]. In the early or latent stages of the disease (namely initial granuloma in this study), a higher percentage of macrophages were observed, increasing considerably in developed granulomas. The composition of these initial granulomas is related to the initial host immune response [5, 11]. The high number of macrophages in the initial stages matches with that observed in early stages in other species such as wild boar [10] and fallow deer [9], although the former also presented a high percentage of T lymphocytes, not observed in sheep. This would suggest that the initial immune response in fallow deer is established by the combined and balanced action of T lymphocytes and macrophages, reducing their presence as the granuloma develops [9]. In sheep and wild boar the presence of macrophages was much greater than T lymphocytes, so the initial immune response in both species would be based on the phagocytosis and lysis of mycobacteria, which is the hallmark of an innate immune response. This, in turn, suggests that sheep response against Mbovis is biased towards the innate unspecific immune response represented by phagocytic cells instead of a well mounted adaptive specific immune response [5]. We have observed a shift towards an adaptive immune response in the more advanced phases of local response, where the larger presence of mycobacteria, showed using Ziehl-Neelsen stain, would drive a humoral rather than cellular immune response. This could lead to a stronger ability to clear initial colonization by small amounts of Mbovis and thus less chance of developing lesions and disease in the absence of an abundant source of Mbovis. The progression to active TB differs among individuals and even among granulomas in a single individual [11]. Before tubercle bacilli can be destroyed by macrophages, these cells must be activated by T lymphocytes and their cytokines, being the essence of cell-mediated immunity [11, 12]. Non-activated macrophages, however, would serve as sites where bacteria are protected within the nascent granuloma [11].

The number of B lymphocytes increased considerably as the granuloma developed. In terminal granulomas, the amount of macrophages decreased while B-lymphocytes increased, suggesting a humoral-mediated immune response. A calcified granuloma (terminal granuloma in this manuscript) generally represents a successful immune response and is associated with fewer inflammatory cells than other granulomas [11]. In the early stages, B cells appeared dispersed and in low quantity. However, in final stages they appeared in large numbers peripherally located, surrounding the caseous necrosis and mineralization, organized in clusters following the same pattern as in studies carried out in cattle [6, 13]. In cattle experimentally challenged with Mbovis, B cell accumulation has been observed together with increasing of chemokines which might suggest non-specific recruitment into lesions rather than a specific humoral response, although more studies are needed to confirm this hyphotesis [13]. In badgers, these final stages are characterized by a large presence of T lymphocytes and scarce B lymphocytes [8], suggesting that the cellular immune response is more important in the elimination of the pathogen in this species than in the sheep. Plasma cells however were more abundant in developed granulomas, as occurs in other species such as badger [8]. These cells appear after activation of B lymphocytes by Th2 lymphocytes and release specific antibodies for antigen destruction [14]. This suggests that an ineffective humoral immune response is the host’s answer to large amounts of antigen accumulated in the granulomata when the unspecific and cellular clearing of mycobacteria has failed. It would be of great interest to carry out subsequent studies in order to increase the sample size and quantify the expression of cytokines such as interferon-gamma (IFN-γ), iNOS or IL-17A in each type of granuloma in order to study the immune responses that accompany the progression of the infection, however, this was not possible in this study because of the excessive fixation time in the processing of the samples.

Lambs subjected to experimental challenge with *M. caprae* showed that the volume of gross pulmonary lesions, quantified by computed tomography and bacterial load in respiratory lymph nodes were similar to those observed in goats experimentally challenged with *M. caprae* at a similar dose suggesting that the susceptibility of sheep to TB infection is similar to that observed in goats [15]. In the present study the number of AFBs within developed and terminal granulomas was very low [1]. This finding would be common in cattle [16, 17] but not in goats with TB infections, where the presence of mycobacteria is higher in these types of lesions in natural cases [17]. Sheep might be considered as a host with a particular and unusual TB lesion development, with low presence of mycobacteria in lesions, indicating the presence of a negative environment for bacterial growth and less capacity of excretion of mycobacteria and, consequently, of spread and transmission [1]. More studies are needed to confirm this hypothesis.

## Conclusions

In conclusion the three types of granulomas described could be the result of different combinations of the three immune response routes: innate, adaptive cellular and adaptive humoral [5]. Thus in the early or latent stages of tissue infection, the innate component would be the most prominent and efficient. If that fails to contain the Mbovis infection, the next step would be an increase in the number of macrophages that would attract more lymphocytes to the site of infection. Finally, a shift to a new phase in the immune response would occur where B lymphocytes and plasma cells would try to contain the infection with a humoral component that would not control the progression and may cause more local damage, as observed in sheep. All these observations could be very useful for identifying factors that may help us to understand the keys that lead to the reactivation of the disease or the persistence of latent infections [18].

## Abbreviations

Ab: Antibody
AFBs: Acid fast bacilli
BSA: Bovine serum albumin
DAB: 3,3′-diaminobenzidine tetrahydrochloride
IHC: Immunohistochemistry
Mbovis: *Mycobacterium bovis*
MTBC: *Mycobacterium tuberculosis* complex
RT: Room temperature
TB: Tuberculosis
TBS: Tris buffered saline
